# Association of childhood leukaemia with factors related to the immune system

**DOI:** 10.1038/sj.bjc.6690395

**Published:** 1999-05-01

**Authors:** J Schüz, U Kaletsch, R Meinert, P Kaatsch, J Michaelis

**Affiliations:** Institut für Medizinische Statistik und Dokumentation der Johannes Gutenberg-Universtität Mainz, D-55101 Mainz, Germany

**Keywords:** case-control study, childhood leukaemia, Germany, immune system, infections

## Abstract

The childhood peak of common acute lymphoblastic leukaemia has been proposed as being a rare response to delayed exposure to a common infection. In this context, factors related to the child’s immune system are of special interest. Information on such factors was obtained in a recent German case-control study comprising more than 1000 children with acute leukaemia. Neither being the first-born child, nor a short duration of breastfeeding, indicators of a deficit in viral contacts during infancy or the number of infectious diseases, were significant risk factors. We observed a strong association with fewer routine immunizations with a 3.2-fold increase for those children getting less than four immunizations, but this association could partly be explained by reporting bias. While tonsillectomy or appendectomy increased the risk of leukaemia in our studies, a protective effect of allergies could be seen. In summary, we found only weak support for the delayed exposure hypothesis. To some extent this may be due to the chosen surrogate markers which reflect, rather indirectly, immunological isolation in infancy and delayed exposure to common viruses. However, the significant findings for routine immunizations, tonsillectomy and allergies of the child or its parents merit further investigation. © 1999 Cancer Research Campaign


*British Journal of Cancer* (1999) **80**(3/4), 585–590
© 1999 Cancer Research Campaign
Article no. bjoc.1998.0395

### Association of childhood leukaemia with factors related to the immune system

**J Schüz, U Kaletsch, R Meinert, P Kaatsch and J Michaelis**

Institut für Medizinische Statistik und Dokumentation der Johannes Gutenberg-Universtität Mainz, D-55101 Mainz, Germany

**Summary** The childhood peak of common acute lymphoblastic leukaemia has been proposed as being a rare response to delayed exposure to a common infection. In this context, factors related to the child's immune system are of special interest. Information on such factors was obtained in a recent German case-control study comprising more than 1000 children with acute leukaemia. Neither being the first-born child, nor a short duration of breastfeeding, indicators of a deficit in viral contacts during infancy or the number of infectious diseases, were significant risk factors. We observed a strong association with fewer routine immunizations with a 3.2-fold increase for those children getting less than four immunizations, but this association could partly be explained by reporting bias. While tonsillectomy or appendectomy increased the risk of leukaemia in our studies, a protective effect of allergies could be seen. In summary, we found only weak support for the delayed exposure hypothesis. To some extent this may be due to the chosen surrogate markers which reflect, rather indirectly, immunological isolation in infancy and delayed exposure to common viruses. However, the significant findings for routine immunizations, tonsillectomy and allergies of the child or its parents merit further investigation.

**Keywords:** case-control study; childhood leukaemia; Germany; immune system; infections

Despite intensified research during the last decades, information regarding the aetiology of primary acute leukaemia during childhood is still limited. Only genetic factors like Down's syndrome, being a homozygotic twin of a child with leukaemia and ionizing radiation are well established as being causal (Pui, 1995). However, these risk factors account only for a small proportion of childhood leukaemias, leaving over 90% of all cases with unidentified aetiological mechanisms so far. Recently, and especially in the UK, it has been hypothesized that the timing of exposure to common infectious agents is of relevance in the aetiology of acute leukaemia (Greaves, 1997). In particular, the risk of developing the major subtype of childhood leukaemia – common acute lymphatic leukaemia (common-ALL), accounting for the age-specific incidence peak of childhood leukaemia at 2–5 years – is suggested to increase with a delayed exposure to a common infectious agent (Alexander, 1997). Some support is available from previous epidemiological studies which have been reviewed by Greaves and Alexander (1993).

The theory that common-ALL around the childhood peak arises as a consequence of an abnormal response to a common infection was first proposed by Greaves (1988, 1997). According to this model, lack of early exposure to common or endemic infections and absence of antigenetic challenge during infancy leaves the immune system of the infant inappropriately modulated. If the child is exposed to a common virus a few years later, its insufficient immunological repertoire could lead either to an inadequate protective immune response or a hyperreactive, unbalanced response of its immune system. The latter reaction could result in proliferative stress, which may promote pre-existing mutant B lymphocyte stem cells that have arisen spontaneously, probably already in utero. This proliferative stress may convert a subclinical pre-leukaemic state into an acute leukaemia. According to Greaves (1997), testable predictions that follow from his theory are that risk of common-ALL should increase with reduced viral contacts in infancy and absence of prolonged breastfeeding. Furthermore, fewer routine immunizations perhaps result in insufficient stimulation of the child's immune system.

In a previous case-control study of childhood leukaemia in the northwestern part of Germany (Lower Saxony) conducted during the years 1992–1995 (Kaatsch et al, 1996; Meinert et al, 1996; Michaelis et al, 1997) we already examined the association between leukaemia and factors related to infections. Based on 173 children with acute leukaemia and 433 control children we found a tendency towards an elevation in risk for first-born children and for children with at most one infectious disease during childhood. The only significantly elevated risk estimate was observed for a fewer number of routine immunizations (three or fewer). Those observations were interpreted as a weak support for Greaves' hypothesis. However, because of the small study population, the risk estimates lacked precision and we could not perform analyses for morphological and immunological subtypes of acute leukaemia. Therefore the investigation was extended to a large German case-control study comprising more than 1000 children with acute leukaemia.

### MATERIALS AND METHODS

During the years 1992–1997, a comprehensive case-control study on childhood cancer and a variety of potential risk factors was conducted in Germany (Kaatsch et al, 1998). Cases were recruited from the nationwide German Children's Cancer Registry at the University of Mainz. This registry was built up in 1980 and is now estimated to be more than 95% complete (Kaatsch et al, 1995). This population-based case-control study consisted of two parts.

*Received 1 May 1998*
*Revised 30 October 1998*
*Accepted 11 November 1998*

*Correspondence to:* J Schüz

**Table 1** Distributions of study setting, gender, age, SES and degree of urbanization for children with acute leukaemia and their corresponding controls (1:1-matching) and for children with common acute lymphatic leukaemia (common-ALL) and all control children (m:n-matching)

| | | 1:1-matching | | | | m:n-matching | | | |
|---|---|---|---|---|---|---|---|---|---|
| | | Leukaemias | | Controls | | common-ALL | | Controls | |
| | | *n* | % | *n* | % | *n* | % | *n* | % |
| Study setting | NW-study only | 561 | 55.5 | [b] | | 379 | 55.2 | 1,900 | 73.4 |
| | NI-study only | 347 | 34.6 | | | 236 | 34.4 | 531 | 20.5 |
| | both parts[a] | 102 | 10.1 | | | 71 | 10.3 | 157 | 6.1 |
| Gender | Male | 598 | 59.2 | [b] | | 394 | 57.4 | 1,504 | 58.1 |
| | Female | 412 | 40.8 | | | 292 | 42.6 | 1,084 | 41.9 |
| Age-group | 0–4 years | 598 | 59.2 | 597 | 59.1 | 447 | 65.2 | 1,359 | 52.5 |
| | 5–9 years | 286 | 28.3 | 287 | 28.4 | 178 | 25.9 | 768 | 29.7 |
| | 10–14 years | 126 | 12.5 | 126 | 12.5 | 61 | 8.9 | 461 | 17.8 |
| SES | Average/low | 767 | 75.9 | 713 | 70.6 | 521 | 75.9 | 1,840 | 71.1 |
| | High | 243 | 24.1 | 297 | 29.4 | 165 | 24.1 | 748 | 28.9 |
| Degree of urbanization | Rural | 292 | 28.9 | [b] | | 194 | 28.3 | 711 | 27.5 |
| | Mixed | 355 | 35.1 | | | 242 | 35.3 | 872 | 33.7 |
| | Urban | 363 | 35.9 | | | 350 | 36.4 | 1,005 | 38.8 |

[a]Comprises children who fulfilled the eligibility criteria of both parts of the study; they were used twice in part-specific analyses but only once in the combined analyses. [b]Because cases and controls were matched for gender, date of birth (1 year) and district, no differences occurred concerning these characteristics.

Both parts comprised children with acute leukaemias, divided into ALL and acute non-lymphatic leukaemia (ANLL). For ALL, we also distinguished the following immunological subtypes: common-ALL, pre-B-ALL, T-ALL and other subtypes (pre-pre-B-ALL, B-ALL and unknown subtype).

For the first part of the study, children aged 14 or less with acute leukaemia were eligible, if the disease was diagnosed between October 1992 and September 1994 and if the child lived in West Germany at the date of diagnosis. This part will be abbreviated the NW-study throughout the text (nationwide part).

The second part of the case-control study was embedded in an ecological study on the incidence of childhood leukaemias and non-Hodgkin's lymphomas in the vicinity of German nuclear installations and selected control regions (Michaelis et al, 1992). Children with acute leukaemia were eligible if the disease occurred between January 1980 and September 1994, if the child was 14 years or younger at date of diagnosis, if the child was born after 1 July 1975, and if the child was living at most 15 km away from a nuclear installation or living in a matched control region at date of diagnosis. This study will be abbreviated the NI-study throughout the text (nuclear installations part). Children with leukaemia fulfilling the eligibility criteria of both parts were considered only once in the combined analyses.

For both parts of the study, controls were gathered by the same sampling protocol. Non-diseased children were randomly selected from complete files of local offices for registration of residents. These files permit the sampling of individuals, even children, and are therefore an adequate frame for population-based studies. For each case, the registration office of the district where the case lived at the date of diagnosis selected one child matched for date of birth and gender. If the chosen family did not participate, another control family was sampled. Therefore the number of contacted controls exceeds the number of contacted cases. Since, due to the German legislation on registration of residents, historical registration files were not accessible, we could only select current controls. While potential controls who were not resident in the sampling area at the time of the case diagnosis could be excluded, eligible children who had subsequently moved out of the sampling area were not included in the sampling frame.

Information on potential risk factors was collected by both questionnaire and telephone interview. The questions were based on a structured questionnaire developed by the US Children's Cancer Group (Buckley et al, 1994). Regarding factors related to the immune system and especially to test the hypothesis by Greaves, it comprised details on birth order, duration of breast-feeding, infectious diseases before the date of diagnosis and which routine immunizations had been performed. The questionnaire listed 18 infectious diseases (those shown in Table 3 and diphtheria, tetanus, poliomyelitis, croup, herpes labialis, rheumatic fever, hepatitis, Pfeiffer's disease and Sticker's disease) and nine routine immunizations (diphtheria, tetanus, pertussis, measles, meningitis, mumps, rubella, poliomyelitis and smallpox) and open categories for other infections or immunizations. We had to estimate a deficit in infant viral contacts by a surrogate criterion. Petridou et al (1993) observed a reduced leukaemia risk for day care attendance of children before the age of three. Since at that time our study was already in progress, we did not ask directly for day care attendance. Therefore we assumed that children were likely to have attended a day care centre if during the first two years of their life both parents were in full-time work (at least half a year at the same time). Additional items of immunological relevance were tonsillectomy or appendectomy in the child and allergies of the child or its parents.

We applied two models for calculating risk estimates. For total leukaemia we used a conditional logistic regression model to obtain the maximum likelihood estimate of the odds ratio (OR) and its 95% confidence interval (CI) with each case matched to its corresponding control (1:1-matching) and further adjustment for socioeconomic status (SES: average, high) estimated by family income and parental education. ORs were also calculated separately for the NW- and the NI-part of the study, but those results are only mentioned if they revealed major study-specific differences.

The second model was used to increase statistical power to detect any association when analyses were based on the

*© Cancer Research Campaign 1999*

**Table 2** Association of total acute leukemia and of common-ALL with factors related to the child's immune system

| | Acute leukaemias | | | Common-ALL | | |
|---|---|---|---|---|---|---|
| | Cases/controls | OR[a] | 95% CI | Cases/controls | OR[b] | 95% CI |
| First-born child | | | | | | |
| No | 493/517 | 1.0 | | 332/1,323 | | |
| Yes | 503/479 | 1.1 | (0.9–1.3) | 348/1,254 | 1.1 | (1.0–1.4) |
| Duration of breastfeeding | | | | | | |
| > 6 months | 146/175 | 1.0 | | 98/421 | 1.0 | |
| 2–6 months | 416/417 | 1.2 | (0.9–1.5) | 294/1,102 | 1.2 | (0.9–1.6) |
| At most 1 month | 439/409 | 1.2 | (0.9–1.6) | 290/1,051 | 1.3 | (1.0–1.7) |
| Deficit in social contacts[c] | | | | | | |
| No | 310/322 | 1.0 | | 226/787 | 1.0 | |
| Yes | 611/599 | 1.1 | (0.9–1.3) | 432/1,468 | 1.0 | (0.8–1.2) |
| Routine immunizations | | | | | | |
| More than 6 | 341/458 | 1.0 | | 255/1,196 | 1.0 | |
| 4–6 | 501/464 | 1.5 | (1.3–1.9)[d] | 343/1,155 | 1.6 | (1.3–1.9)[d] |
| 0–3 | 150/70 | 3.2 | (2.3–4.6)[d] | 80/228 | 2.1 | (1.5–2.9)[d] |
| Infections | | | | | | |
| More than one | 551/571 | 1.0 | | 372/1,461 | 1.0 | |
| At most one | 443/423 | 1.1 | (0.9–1.3) | 310/1,105 | 1.0 | (0.8–1.2) |
| Tonsillectomy/appendectomy | | | | | | |
| None | 761/796 | 1.0 | | 564/2,104 | 1.0 | |
| At least one | 146/111 | 1.4 | (1.1–1.9)[d] | 87/352 | 1.1 | (0.8–1.5) |
| Allergy of the child | | | | | | |
| No | 908/859 | 1.0 | | 620/2,220 | 1.0 | |
| Yes | 93/142 | 0.6 | (0.5–0.8)[d] | 61/366 | 0.6 | (0.5–0.9)[d] |
| Allergy of the mother | | | | | | |
| No | 819/779 | 1.0 | | 559/2,003 | | |
| Yes | 182/222 | 0.8 | (0.6–1.0)[d] | 122/583 | 0.7 | (0.6–0.9)[d] |

[a]Derived from a 1:1-matched conditional logistic regression model (adjusted for SES). [b]Derived from a m:n-matched conditional logistic regression model stratified for gender, age, year of birth, and study setting, and adjusted for SES and degree of urbanization. [c]Only children aged older than 18 months. [d]$P < 0.05$.

morphological and immunological subgroups of acute leukaemias. The control group comprised all available control children of both parts of the study for all cancers even if the individual match had not participated. Therefore for common-ALL the ORs were calculated using frequency matching [m:n-matching (cases:controls)] in a conditional logistic regression model (Brookmeyer et al, 1986). We performed a posteriori-stratification for gender, age (age-groups of 1 year), year of birth and vicinity to a nuclear installation (yes, no). Additional adjustments for degree of urbanization (rural, mixed, urban) and SES were made.

### RESULTS

In all, 1184 families of children with leukaemia and 2588 families with control children participated in the case-control study. In the NW-study, 82.2% of all case families who received the questionnaire sent it back to the study centre. Regarding the NI-study, the response was slightly lower (80.0%). The response rates for control families were 68.6% for the NW-study and 61.6% for the NI-study respectively. Telephone interviews were performed with 95.1% of the participating parents of children with leukaemia and with 95.2% of the participating parents of controls. More than two-thirds of all non-participants were refusals. Other reasons for non-participation were lost to follow-up, violations of the eligibility criteria and insufficient knowledge of the German language. A total of 174 cases with leukaemia had to be excluded from the 1:1-matched analyses, because we failed to recruit a corresponding control. The 1010 children with acute leukaemia available for the 1:1-matched regression analyses break down into 588 cases with common-ALL (58.2%), 106 cases with pre-B-ALL (10.5%), 87 cases with T-ALL (8.6%), 103 cases with ALL of other or unknown subtype (10.2%) and 126 cases with ANLL (12.5%). Using the m:n-matched regression analyses their numbers increase to 686 for common-ALL, 121 for pre-B-ALL, 99 for T-ALL, 131 for ALL of other subtype and 147 for ANLL respectively. Table 1 shows the demographic information for cases and controls available for the 1:1- and m:n-matched analyses. Since children with acute leukaemia and their corresponding controls were matched for gender, date of birth and district, they only differ with regard to SES. Because the NW-study also comprised other types of tumours, the number of controls for the m:n-matched analyses is almost four times the number of controls of the NI-study.

Table 2 shows the main results from the analyses, as described in the Methods section. Birth order was very weakly associated with leukaemia; regarding common-ALL the risk for first-born children was of borderline statistical significance. Twins without older siblings were considered as being first-born.

Absence of prolonged breastfeeding was weakly associated with leukaemia. This was also the case for children who were breastfed for 2–6 months; for children who were breastfed for no more than 1 month the ORs were only moderately elevated. However, regarding common-ALL the elevation in risk was of borderline statistical significance.

**Table 3** Association of total acute leukaemia with infectious diseases

| | Unexposed | | Exposed | | | |
|---|---|---|---|---|---|---|
| | Cases | Controls | Cases | Controls | OR[a] | 95% CI |
| Chickenpox | 590 | 552 | 403 | 441 | 0.8 | (0.7–1.0)[b] |
| Measles | 909 | 911 | 101 | 99 | 1.0 | (0.7–1.4) |
| Mumps | 966 | 954 | 43 | 56 | 0.7 | (0.5–1.5) |
| Rubella | 887 | 886 | 87 | 88 | 1.0 | (0.7–1.4) |
| Scarlet fever | 862 | 847 | 114 | 129 | 0.8 | (0.6–1.1) |
| Pneunomia | 895 | 916 | 88 | 67 | 1.3 | (1.0–1.9) |
| Bronchitis | 567 | 592 | 386 | 361 | 1.1 | (0.9–1.4) |
| Pertussis | 800 | 793 | 181 | 188 | 1.0 | (0.7–1.2) |
| Inflammation of the middle ear | 611 | 614 | 358 | 355 | 1.0 | (0.8–1.2) |

[a]Derived from a 1:1-matched conditional logistic regression model (adjusted for SES). [b]$P < 0.05$.

Our surrogate for a deficit in social contacts (and therefore deficit of viral contacts) with other children revealed ORs close to unity for total leukaemia and for common-ALL respectively. The results did not change if these analyses were restricted to first-born children (data not shown).

Children with fewer routine immunizations had a higher risk of developing a leukaemia compared to children who were immunized against six diseases or more. We observed a dose–response relationship as shown in Table 2. The association was even more pronounced for children aged 3 years or less: for the category of 0–3 immunizations we observed a 10.5-fold increase (95% CI: 5.1–21.7) based on 72 leukaemia cases and 17 controls. Consequently, for children aged 4 years or more, the risk estimates were somewhat lower, but still statistically significantly increased (4–6 immunizations: OR 1.3 (95% CI: 1.0–1.7), 361 cases, 355 controls; 0–3 immunizations: OR 1.8 (95% CI: 1.2–2.7), 73 cases, 53 controls].

Infections during childhood did not seem to be associated with the incidence of leukaemia in our study. For children having no or one infectious disease before the date of diagnosis, the ORs were close to unity. The leukaemia risk for children aged 1.5 years or older at date of diagnosis who had no infectious disease during the first year of life was 0.9 (95% CI: 0.7–1.1) regarding all leukaemia cases and 1.0 (95% CI: 0.8–1.2) regarding common-ALL; for those who had at least one infectious illness the year before diagnosis the ORs were 1.1 (95% CI: 0.9–1.4) for total leukaemia and 1.2 (95% CI: 1.0–1.5, $P > 0.05$) for common-ALL respectively. Following the concept that absence of exposure to infection in the first year of life along with exposure to an infectious agent later increases risk, we estimated the risk for children aged 2 years or older who had no infectious disease during the first year of life but at least one infectious disease during the year preceding diagnosis compared to those who had at least one infectious illness during the first year of life or no infectious illness the year before diagnosis or both. The OR was 1.0 (95% CI: 0.8–1.3) for total leukaemia and 1.2 (95% CI: 1.0–1.5, $P > 0.05$) for common-ALL respectively.

Table 3 shows the results for the different infectious diseases (only infections with a prevalence of more than 2% are shown). We observed a significant association with chickenpox. Regarding common-ALL, the OR for chickenpox did not remain statistically significant (OR 0.9, 95% CI: 0.7–1.1, 261 cases, 1197 controls). Instead, we found a statistically significant increase in risk for pneumonia (OR 1.7, 95% CI: 1.2–2.3) based on 66 cases and 167 controls. If only children with an infection the year before the reference date (cases: date of diagnosis, controls: date of diagnosis of the corresponding case) were considered as being exposed, the ORs were only slightly changed except for bronchitis and pneunomia. Here we observed elevated risk estimates of 2.4 (95% CI: 1.5–3.9, bronchitis) and 2.1 (95% CI: 1.1–3.8, pneunomia) for total leukaemia, and ORs of 1.9 (95% CI: 1.3–2.7, bronchitis) and 2.6 (95% CI: 1.4–4.8, pneunomia) for common-ALL respectively. Those calculations were restricted to children aged 18 months or older.

Table 2 also shows the ORs for the hypothesis that children who had a tonsillectomy or appendectomy had a higher risk of developing an acute leukaemia. Concerning the immunological and morphological subtypes of leukaemia, the ORs are highest for T-ALL (OR 1.5, 95% CI: 0.9–2.6, 28 cases, 352 controls) and ANLL (OR 1.5, 95% CI: 0.9–2.6, 26 cases, 352 controls), while for common-ALL and pre-B-ALL they are close to unity. With a prevalence of approximately 1%, appendectomy contributes only little to the combined risk estimates. Treatment-specific analyses reveal a statistically significantly increased OR of 1.4 for tonsillectomy (95% CI: 1.0–1.9) based on 136 cases and 107 controls and a relatively unprecise OR of 1.7 for appendectomy (95% CI: 0.6–4.2) based on only 12 cases and seven controls.

Our results indicate that an allergy may be protective against acute childhood leukaemia. For both allergies of the child and the parents the ORs are below unity; however, for paternal allergies the association is not statistically significant (OR 0.8, 95% CI: 0.6–1.1, 115 cases, 140 controls). For allergies of the child there were no major differences between the morphological and immunological subtypes of acute leukaemia with ORs ranging between 0.5 and 0.9, but the risk estimate derived from the NI-study was considerably lower (OR 0.3, 95% CI: 0.2–0.6) than that derived from the NW-study (OR 0.9, 95% CI: 0.6–1.3). In addition to that, the risk for children receiving no medicinal treatment of their allergy was lower (OR 0.5, 95% CI: 0.4–0.7) than that for children being medicinally treated (OR 0.9, 95% CI: 0.6–1.4). For allergies of the mother there were no differences concerning the two different study parts. Regarding the immunological subtypes, the ORs were statistically significantly decreased for common-ALL as well as for T-ALL (data not shown).

### DISCUSSION

One strength of the German case-control study is that it was population-based, because cases were identified by an almost complete

*© Cancer Research Campaign 1999*

nationwide cancer registry and controls were drawn at random from complete files of population registries. The response rates were very good and a study population comprising more than 1000 children with acute leukaemia allows analyses for the most common immunological subtypes of ALL. Controls were matched for gender, age and district. Because families with control children of higher average monthly family income were more likely to participate than those with lower income, we additionally adjusted for SES in all analyses. However, since population files are not available historically, selection bias cannot completely be ruled out.

The two parts of our study differ regarding the periods of diagnosis and one part was restricted to specific geographical areas. The study designs and the procedures of control selection of the two parts were equal; we used the same questionnaire and the interviews were done by the same trained interviewers. Since the recall period for participants of the NI-study was considerably longer, all ORs were also calculated separately for both parts of the study and we compared the prevalence of each risk factor regarding part-specific variations. Regarding the immunological subtypes of leukaemia, we preferred a frequency-matched logistic regression model to the individual 1:1-matching, so the results are based on larger numbers and are less likely to be affected by coincidental differences in the prevalences of risk factors among small groups of control children. However, for these analyses, cases are more likely than controls to have the reference date in the period of 1980–1991 (but analyses were stratified for year of birth and age at date of diagnosis) and there was no individual matching for date of diagnosis and hence season of diagnosis.

Our interviewers were trained regularly and the course of the interview was given in detail. Nevertheless, they could not be blinded to case-control status of the participating family. All information on exposure was assessed by questionnaire and telephone interview and might therefore be influenced by recall bias. For the NW-study, the interviews were performed short time after the date of diagnosis, but regarding the NI-study the time interval between the date of diagnosis and the interview date might have been up to 15 years.

In particular, regarding routine immunizations, we found evidence of differential reporting bias. As immunizations against measles, mumps and rubella are only recommended after the 15th month of the child's life, we expect that very few children aged one year or younger were immunized against these diseases. But while, for example, 3% of all parents of children for whom the leukaemia was diagnosed before the age of one reported immunization against measles, a surprising 54% of parents of all control children aged one or younger stated that their child had been immunized against measles. Obviously, parents of control children would more often mistakenly report immunizations which, in fact, had been given after the reference date. The results for allergies of the child might have been biased in a similar way, if we assume that parents also mentioned allergies which occurred after the reference date. Because of the immunosuppressive treatment, children with leukaemia will be less likely to develop an allergy than non-diseased children and therefore, after the reference date, allergies occur more often in control children.

The large number of comparisons could result in some statistically significant findings which might have occurred by chance. We did not adjust for multiple testing. Instead, we discuss an individual finding taking into account its consistency with previous international studies and its aetiological background.

Birth order was only weakly associated with acute leukaemia, which accords with other recent investigations (Petridou et al, 1997; Robison et al, 1987; Ross et al, 1997; Shu et al, 1988). Earlier studies had observed a leukaemia risk for first-born children (McMahon and Newill, 1962; Stark and Mantel, 1966; Van Steensel-Moll et al, 1986). In agreement with other investigations, the duration of breastfeeding was not significantly associated with leukaemia risk (Hartley et al, 1988; Van Duijn et al, 1988; Shu et al, 1995; Petridou et al, 1997). However, for common-ALL the elevation in risk was of borderline statistical significance. Our surrogate for a deficit in social contacts with other children seems to represent immunological isolation during infancy only vaguely. Better indicators of viral contacts during infancy are necessary to test the prediction that an insufficiently modulated infant immune system and delayed exposure to one or more common infections increases the leukaemia risk of the child. Virus infections were not associated with childhood leukaemia in our study. Distinguishing between the infections, we found a risk reduction for chickenpox for all leukaemias and an elevated risk for pneunomia for common-ALL. A negative association between infections in the first year of life and childhood leukaemia was reported by Van Steensel-Moll et al (1986). We could not confirm this observation in our studies. The hypothesis of a delayed exposure to a common infection implies that exposure to infections has a protective effect during infancy but increases the leukaemia risk some time later. Early and delayed infections, especially for younger children, are not readily told apart and infections with a subclinical course or only minor symptoms cannot be identified. For these reasons our risks estimates might have been biased towards the null value. We found somewhat elevated risks for children who had at least one infectious disease the year before diagnosis. In this context, the striking results for bronchitis and pneunomia occurring in the year before the reference date might be a weak indicator for a specific infectious agent involved in the delayed exposure hypothesis (Alexander, 1997). Another possible explanation is that these infections are prodromal manifestations of the malignancy. However, these findings might have been influenced by recall bias or might be due to chance.

In summary, our results provide only weak support for Greaves' hypothesis and therefore only partially confirmed the results of our previous study in Lower Saxony. Part of the association between fewer routine immunizations and leukaemia can be explained by reporting bias as mentioned above and by prodromal manifestations of the malignancy preventing the conduct of regular immunization schemes. Previous studies have been inconsistent on this topic, while some found evidence that immunizations convey protection (Kneale et al, 1986; Hartley et al, 1988; Nishi and Miyake, 1989), this was not confirmed by recent studies (Buckley et al, 1994; Petridou et al, 1997). Hence, if a negative association between routine immunizations and the occurrence of childhood acute leukaemia exists, we assume it to be weak as implied by the risk estimates for older children. Additional analyses of this important question are needed, preferably on the basis of copied vaccination cards instead of questionnaires.

Tonsillectomied or appendectomied children had a higher risk of developing an acute leukaemia in our studies. Possibly the resection of lymphatic tissue and the resulting hampering of the immune system might be a potential aetiological mechanism, but also the resection as a consequence of prior susceptibility to infectious diseases should be considered as an indicator of a biological

© Cancer Research Campaign 1999

link. Zheng et al (1993) reported an association between appendectomy, tonsillectomy and adult leukaemia. If the inverse association between allergies of the child and acute leukaemia, also observed by Nishi and Miyake (1989) and Petridou et al (1997), is not due to reporting bias, the protective effect of allergies could be interpreted as a proxy for a well-functioning, even overreacting immune system. Since for allergies there seems to exist a genetic predisposition and children of parents with allergies tend to develop an allergy (Daniels et al, 1996), the result, for maternal allergies especially, is not surprising and might be less influenced by recall bias than that for allergies of the child. However, in this field, further studies, including immunological laboratory examinations, are needed.

### REFERENCES

Alexander F (1997) Is mycoplasma pneunomia associated with childhood acute lymphoblastic leukemia? *Cancer Causes Control* **8**: 803–811

Brookmeyer R, Liang KY and Linet M (1986) Matched case-control designs and overmatched analyses. *Am J Epidemiol* **124**: 693–701

Buckley JD, Buckley CM, Ruccione K, Sather HN, Waskerwitz MJ, Woods WG and Robison LL (1994) Epidemiological characteristics of childhood acute lymphocytic leukemia. Analysis by immunophenotype. The Children’s Cancer Group. *Leukemia* **8**: 856–864

Daniels SE, Bhattacharrya S, James A, Leaves NI, Young A, Hill MR, Faux JA, Ryan GF, le Souef PN, Lathrop GM, Musk AW and Cookson WO (1996) A genome-wide search for quantitative trait loci underlying asthma. *Nature* **383**: 247–250

Greaves MF (1988) Speculations on the cause of childhood acute lymphoblastic leukemia. *Leukemia* **2**: 120–125

Greaves MF (1997) Aetiology of acute leukaemia. *Lancet* **349**: 344–349

Greaves MF and Alexander FE (1993) An infectious etiology for common acute lymphoblastic leukemia in childhood? *Leukemia* **7**: 349–360

Hartley AL, Birch JM, McKinney PA, Blair V, Teare MD, Carrette J, Mann JR, Stiller CA, Draper GJ and Johnston HE (1988) The Inter-Regional Epidemiological Study of Childhood Cancer (IRESCC): past medical history in children with cancer. *J Epidemiol Community Health* **42**: 235–242

Kaatsch P, Haaf G and Michaelis J (1995) Childhood malignancies in Germany – methods and results of a nationwide registry. *Eur J Cancer* **31A**: 993–999

Kaatsch P, Kaletsch U, Krummenauer F, Meinert R, Miesner A, Haaf G and Michaelis J (1996) Case control study on childhood leukemia in Lower Saxony, Germany. Basic considerations, methodology, and summary of results. *Klin Pädiatr* **208**: 179–185

Kaatsch P, Kaletsch U, Meinert R, Miesner A, Hoisl M, Schüz J and Michaelis J (1998) German case control study on childhood leukemia – basic considerations, methodology, and summary of the results. *Klin Pädiatr* **210**: 185–191

Kneale GW, Stewart AM and Wilson LM (1986) Immunizations against infectious diseases and childhood cancers. *Cancer Immunol Immunother* **21**: 129–132

McMahon B and Newill VA (1962) Birth characteristics of children dying of malignant neoplasms. *J Natl Cancer Inst* **28**: 231–244

Meinert R, Kaatsch P, Kaletsch U, Krummenauer F, Miesner A and Michaelis J (1996) Childhood leukaemia and exposure to pesticides: results of a case-control study in northern Germany. *Eur J Cancer* **32A**: 1943–1948

Michaelis J, Keller B, Haaf G and Kaatsch P (1992) Incidence of childhood malignancies in the vicinity of west German nuclear power plants. *Cancer Causes Control* **3**: 255–263

Michaelis J, Schüz J, Meinert R, Menger M, Grigat JP, Kaatsch P, Kaletsch U, Miesner A, Stamm A, Brinkmann K and Kärner H (1997) Childhood leukemia and electromagnetic fields: results of a population-based case control study in Germany. *Cancer Causes Control* **8**: 167–174

Nishi M and Miyake H (1989) A case-control study of non-T cell acute lymphoblastic leukaemia of children in Hokkaido, Japan. *J Epidemiol Community Health* **43**: 352–355

Petridou E, Kassimos D, Kalmanti M, Kosmidis H, Haidas S, Flytzani V, Tong D and Trichopoulos D (1993) Age of exposure to infections and risk of childhood leukaemia. *Br Med J* **307**: 774

Petridou E, Trichopoulos D, Kalapothaki V, Pourtsidis A, Kogevinas M, Kalmanti M, Koliouskas D, Kosmidis H, Panagiotou JP, Piperopoulou F and Tzortzatou F (1997) The risk profile of childhood leukemia in Greece: a nationwide case-control study. *Br J Cancer* **76**: 1241–1247

Pui C (1995) Childhood leukemias. *N Engl J Med* **332**: 1618–1630

Robison LL, Codd M, Gunderson P, Neglia JP, Smithson WA and King FL (1987) Birth weight as a risk factor for childhood acute lymphoblastic leukemia. *Pediatr Hematol Oncol* **4**: 63–72

Ross JA, Potter JD, Shu X-O, Reaman GH, Lampkin B and Robison LL (1997) Evaluating the relationships among maternal reproductive history, birth characteristics, and infant leukemia: a report from the Children’s Cancer Group. *Ann Epidemiol* **7**: 172–179

Shu XO, Gao YT, Brinton LA, Linet MS, Tu JT, Zheng W and Fraumeni JF (1988) A population-based case-control study of childhood leukemia in Shanghai. *Cancer* **62**: 635–644

Shu XO, Clemens J, Zheng W, Ying DM, Ji BT and Jin F (1995) Infant breastfeeding and the risk of childhood lymphoma and leukaemia. *Int J Epidemiol* **24**: 27–32

Stark CR and Mantel N (1966) Effects of maternal age and birth order on the risk of mongolism and leukemia. *J Natl Cancer Inst* **37**: 687–698

Van Duijn CM, Van Steensel-Moll HA, Van der Does vd Berg A, Van Wering ER, Van Zanen GE, Valkenburg HA and Rammeloo JA (1988) Infant feeding and childhood cancer. *Lancet* **2**: 796–797

Van Steensel-Moll HA, Valkenburg HA, Vandenbroucke JP, Van Zanen GE (1986) Are maternal fertility problems related to childhood leukaemia? *Int J Epidemiol* **14**: 555–559

Zheng W, Linet MS, Shu XO, Pan RP, Gao YT and Fraumeni JF Jr (1993) Prior medical conditions and the risk of adult leukemia in Shanghai, People’s Republic of China. *Cancer Causes Control* **4**: 361–368

 © Cancer Research Campaign 1999
